# Supplementary material for: Antibiotic Prescribing Trends in Belgian Out-of-Hours Primary Care during the COVID-19 Pandemic: Observational Study Using Routinely Collected Health Data
Source: Antibiotics (Basel). 2021 Dec 4;10(12):1488. doi: 10.3390/antibiotics10121488 (PMC8698421; doi:10.3390/antibiotics10121488)
Supplement: Supplementary file 1 [file antibiotics-10-01488-s001.zip › antibiotics-1463119-SI.pdf]

antibiotics

MDPI

Supplemental material

Antibiotic prescribing trends in Belgian out-of-hours primary care during the COVID-19 pandemic: observational study using routinely collected health data

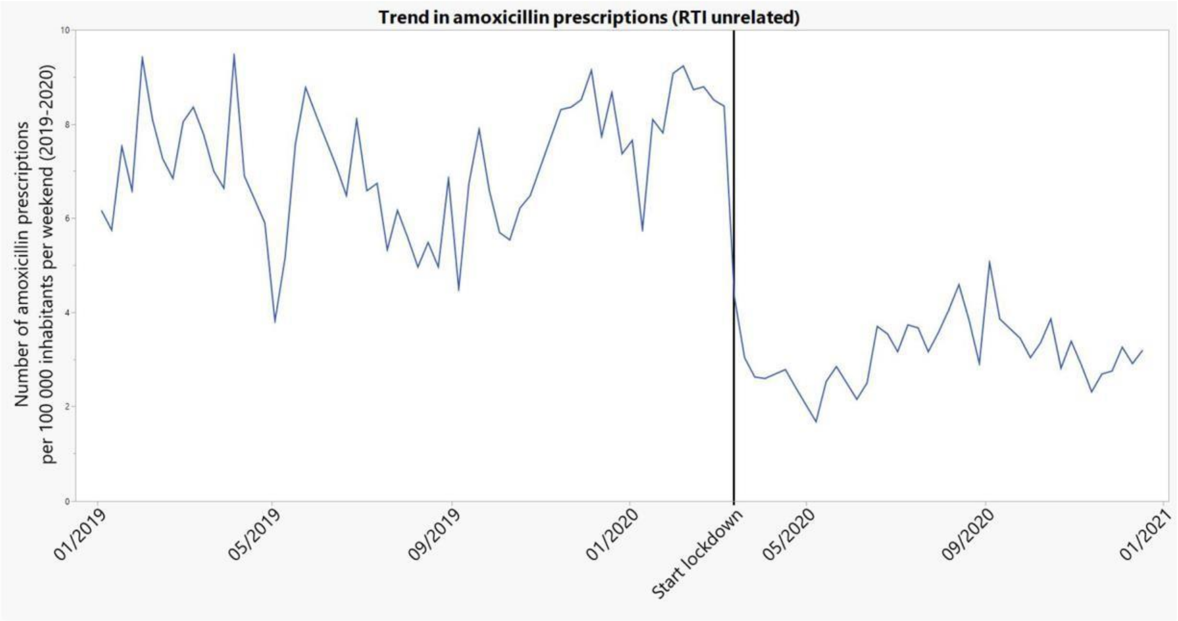

Figure S1: Number of antibiotic prescriptions per 100 000 inhabitants per weekend over time (2019-2020) for RTI unrelated contacts (ICPC codes not directly linked with possible COVID-19 infection, incl. throat/ear infections)

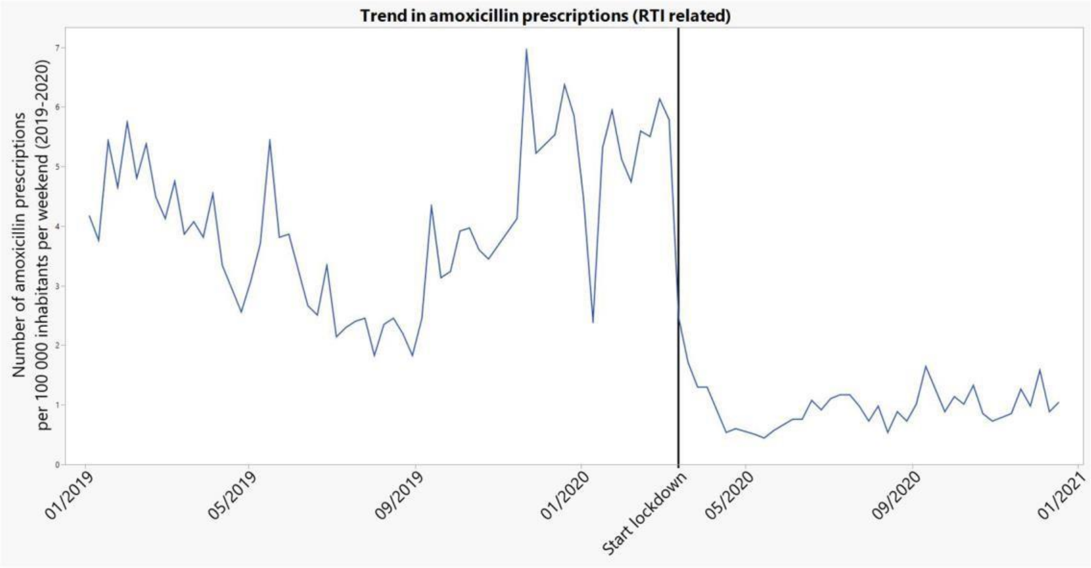

Figure S2: Number of antibiotic prescriptions per 100 000 inhabitants per weekend over time (2019-2020) for RTI related contacts (excl. throat/ear infections) (incl. ICPC codes: “R74: acute upper respiratory tract infection", "R83: other airway infections", "R81: pneumonia", "A77: other viral infections", "A78: other infections", "A03: fever", "R02: dyspnoe", "R05: coughing", "R80: influenza", "R78: acutebronchitis")

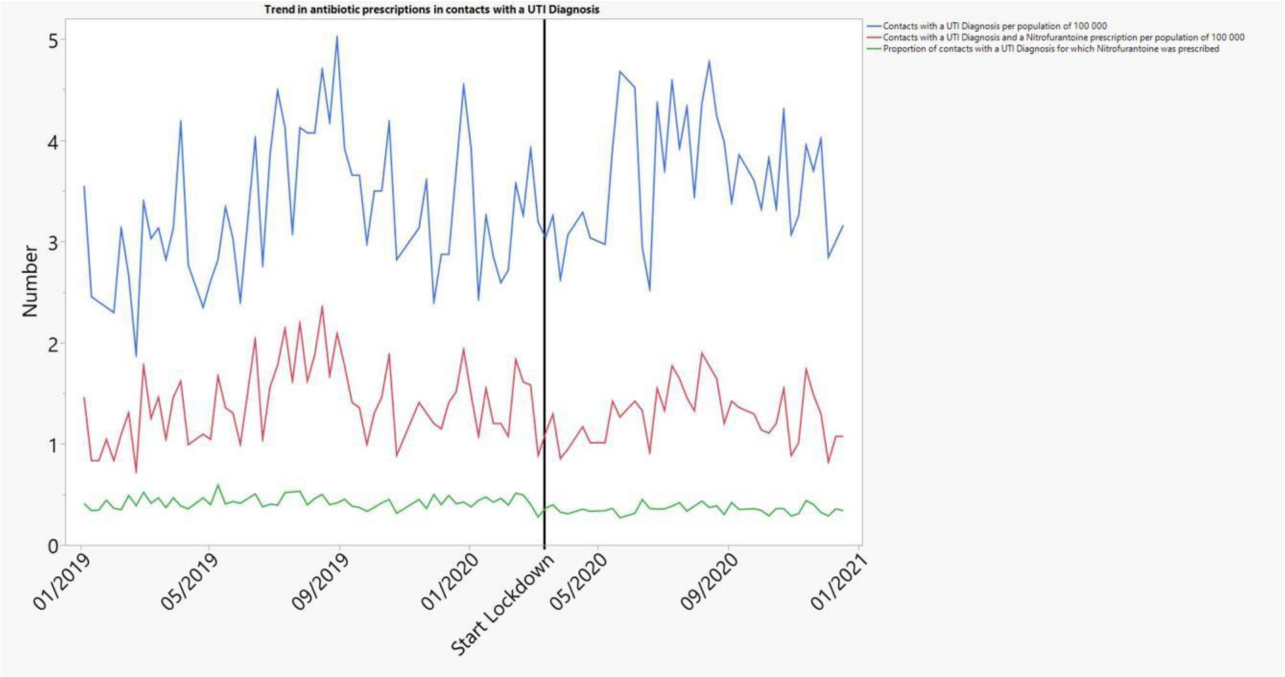

Figure S3. The trend in UTI diagnosis and nitrofurantoin prescribing per 100 000 population

File S1: Description and model fit of the ARIMA models

2.2.1 General trend in antibiotic prescribing

Regression with ARIMA(1,0,0) errors

Coefficients:

	ar1	intercept	xreg
	0.4815	26.7331	-11.6743
s.e.	0.0852	0.6858	1.0566

sigma^2 estimated as 8.419: log likelihood=-254.58

AIC=517.16 AICc=517.57 BIC=527.74

Training set error measures:

	ME	RMSE	MAE	MPE	MAPE	MASE	ACF1
Training set	0.009437571	2.858904	2.237814	-1.83116	10.73982	0.2298484	-0.005751584

2.2.2.a Trends in prescribing of amoxicillin

Regression with ARIMA(5,0,0) errors

Coefficients:

	ar1	ar2	ar3	ar4	ar5	intercept	xreg
	0.5571	0.2476	-0.2289	0.2059	-0.0125	11.3680	-7.3642
s.e.	0.0985	0.1100	0.1137	0.1151	0.1039	0.6654	0.9280

sigma^2 estimated as 1.928: log likelihood=-176.98

AIC=369.96 AICc=371.48 BIC=391.12

Training set error measures:

	ME	RMSE	MAE	MPE	MAPE	MASE	ACF1
Training set	0.01922395	1.340457	0.987874	-2.389671	12.91813	0.1809554	0.00608261

2.2.2.b Trends in prescribing of amoxicillin/clavulanate

Regression with ARIMA(0,0,0) errors

Coefficients:

	intercept	xreg
	5.7715	-2.1539
s.e.	0.1130	0.1791

sigma^2 estimated as 0.807: log likelihood=-134.1

AIC=274.19 AICc=274.43 BIC=282.12

Training set error measures:

	ME	RMSE	MAE	MPE	MAPE	MASE	ACF1
Training set	4.656448e-16	0.8895524	0.7151907	-3.331603	15.40719	0.3596296	0.1052156

2.2.3 Trend in antibiotic prescribing per contact type

a) All contacts:

Regression with ARIMA(1,0,0) errors

Coefficients:

	ar1	intercept	xreg
	0.4629	0.2160	-0.1201
s.e.	0.0870	0.0044	0.0068

sigma^2 estimated as 0.0003677: log likelihood=262.42

AIC=-516.85 AICc=-516.44 BIC=-506.27

Training set error measures:

	ME	RMSE	MAE	MPE	MAPE	MASE	ACF1
Training set	-9.131262e-05	0.018895	0.01411352	-2.157768	10.16329	0.1393096	-0.08143524

b) face-to-face

Regression with ARIMA(4,0,0) errors

Coefficients:

	ar1	ar2	ar3	ar4	intercept	xreg
	0.3022	0.2089	-0.1030	0.0440	0.2168	-0.0519
s.e.	0.0981	0.1023	0.1035	0.0987	0.0045	0.0069

sigma^2 estimated as 0.0004071: log likelihood=258.79

AIC=-503.57 AICc=-502.4 BIC=-485.06

Training set error measures:

	ME	RMSE	MAE	MPE	MAPE	MASE	ACF1
Training set	-6.68857e-05	0.01958014	0.0143292	-1.181269	7.818679	0.3010191	0.004993282

2.2.4 Trends in prescribing of nitrofurantoin

Regression with ARIMA(2,0,1) errors

Coefficients:

	ar1	ar2	ma1	intercept	xreg
	0.6922	0.0894	-0.5540	1.5120	-0.1100

s.e. 0.2728 0.1328 0.2621 0.0809 0.1214

sigma^2 estimated as 0.1134: log likelihood=-31.59
AIC=75.17 AICc=76.04 BIC=91.04

Training set error measures:

	ME	RMSE	MAE	MPE	MAPE	MASE	ACF1
Training set	0.00482198	0.3284207	0.2681592	-4.927788	19.5145	0.7017481	0.004548992
